# Generation of Antigen Microarrays to Screen for Autoantibodies in Heart Failure and Heart Transplantation

**DOI:** 10.1371/journal.pone.0151224

**Published:** 2016-03-11

**Authors:** Andrzej Chruscinski, Flora Y. Y. Huang, Albert Nguyen, Jocelyn Lioe, Laura C. Tumiati, Stella Kozuszko, Kathryn J. Tinckam, Vivek Rao, Shannon E. Dunn, Michael A. Persinger, Gary A. Levy, Heather J. Ross

**Affiliations:** 1 Multi-Organ Transplant Program, University Health Network, Toronto, Ontario, Canada; 2 Division of Cardiology, University Health Network, Toronto, Ontario, Canada; 3 Department of Immunology, University of Toronto, Toronto, Ontario, Canada; 4 Division of Cardiac Surgery, University Health Network, Toronto, Ontario, Canada; 5 Toronto General Research Institute, Toronto, Ontario, Canada; 6 Women’s College Research Institute, Toronto, Ontario, Canada; 7 Behavioral Neuroscience, Biomolecular Sciences and Human Studies Programs, Laurentian University, Sudbury, Ontario, Canada; Weizmann Institute of Science, ISRAEL

## Abstract

Autoantibodies directed against endogenous proteins including contractile proteins and endothelial antigens are frequently detected in patients with heart failure and after heart transplantation. There is evidence that these autoantibodies contribute to cardiac dysfunction and correlate with clinical outcomes. Currently, autoantibodies are detected in patient sera using individual ELISA assays (one for each antigen). Thus, screening for many individual autoantibodies is laborious and consumes a large amount of patient sample. To better capture the broad-scale antibody reactivities that occur in heart failure and post-transplant, we developed a custom antigen microarray technique that can simultaneously measure IgM and IgG reactivities against 64 unique antigens using just five microliters of patient serum. We first demonstrated that our antigen microarray technique displayed enhanced sensitivity to detect autoantibodies compared to the traditional ELISA method. We then piloted this technique using two sets of samples that were obtained at our institution. In the first retrospective study, we profiled pre-transplant sera from 24 heart failure patients who subsequently received heart transplants. We identified 8 antibody reactivities that were higher in patients who developed cellular rejection (2 or more episodes of grade 2R rejection in first year after transplant as defined by revised criteria from the International Society for Heart and Lung Transplantation) compared with those who did have not have rejection episodes. In a second retrospective study with 31 patients, we identified 7 IgM reactivities that were higher in heart transplant recipients who developed antibody-mediated rejection (AMR) compared with control recipients, and in time course studies, these reactivities appeared prior to overt graft dysfunction. In conclusion, we demonstrated that the autoantibody microarray technique outperforms traditional ELISAs as it uses less patient sample, has increased sensitivity, and can detect autoantibodies in a multiplex fashion. Furthermore, our results suggest that this autoantibody array technology may help to identify patients at risk of rejection following heart transplantation and identify heart transplant recipients with AMR.

## Introduction

Autoantibodies directed against heart antigens are often present in patients with heart failure [1]. Studies have demonstrated that some of these autoantibodies are pathogenic and can directly promote cardiac dysfunction. For example, autoantibodies against cardiac myosin and troponin I can induce cardiomyopathies in animal models [2, 3]. Measuring autoantibodies is important as it may help identify which patients are candidates for therapies such as immunoadsorption.

In transplantation, there is evidence that pre-transplant autoimmunity in the form of autoantibodies is associated with more rejection episodes post-transplant. Studies in humans have shown that pre-transplant autoantibodies to cardiac myosin are associated with an increased risk of cellular rejection following heart transplantation [4]. A direct link between pre-transplant autoimmunity and increased risk of rejection has been demonstrated in experimental models of transplantation where pre-transplant immunization with either cardiac myosin or vimentin leads to accelerated rejection following heart transplantation [5, 6]. Detection of autoantibodies may thus be useful in identifying transplant recipients at higher risk of rejection.

After transplant, both immune cells and antibodies can damage allografts, leading to rejection. In cell-mediated rejection, immune cells infiltrate and damage the allograft. Cell-mediated rejection is diagnosed by endomyocardial biopsy and is typically reversed by increasing immunosuppression. If a heart transplant recipient shows evidence of a decline in heart function, but the endomyocardial biopsy is negative for immune cell infiltration, more specialized immunohistochemical stains are performed, including detection of the complement degradation product C4d [7, 8]. If complement deposition is detected or certain pathological changes are noted, antibody-mediated rejection (AMR) is usually suspected. This type of rejection occurs in approximately 10–20% of heart transplant patients, is being increasing recognized as a major cause of morbidity and mortality in heart transplant recipients, and is often difficult to treat, since conventional immunosuppression does not target antibody production [7–9]. AMR is also typically associated with the presence of donor-specific anti-HLA antibodies, which can bind to endothelial cells, initiate the classical pathway of complement, and invoke inflammatory damage on capillary endothelium [10]. More recently, non-HLA antibodies against myosin and vimentin have been identified in the serum of heart transplant recipients with AMR [11]. Importantly, there is evidence that detection of these antibodies may aid in the diagnosis of AMR as their appearance precedes overt graft dysfunction [11].

Detection of autoantibodies can be laborious as each autoantibody is typically measured by performing an ELISA. Since the autoantibodies may differ from patient to patient, many ELISAs need to be performed to capture the breadth of these reactivities, thus consuming a large volume of patient sample. In order to further understand the role of autoantibodies in heart failure and heart transplantation, a more comprehensive method for profiling these antibodies is needed.

Antigen microarrays are a novel, high-throughput technology used for the simultaneous detection of multiple antigen-antibody interactions. In this technique, multiple antigens are spotted onto coated microscope slides using a robotic microarrayer and are probed with antibodies in patient sera, which are then detected using fluorophore-conjugated secondary antibodies [12]. Compared with the traditional ELISA technique, antigen microarrays require lower amounts of reagents and are also two to sixteen-fold more sensitive [12]. In past studies, antigen microarrays have been used for the screening of autoantibodies in various autoimmune diseases including rheumatoid arthritis and multiple sclerosis [13, 14]. This technology has also been used to profile non-HLA antibodies in kidney and lung transplant recipients [15–17].

Here we describe the use of a custom-made antigen microarray for the profiling of antibodies in patients both pre- and post-heart transplantation. We also describe the generation of two-color array technique that allows for the discrimination of IgG and IgM reactivities. In two small retrospective studies, we demonstrate the utility of microarrays for antibody detection in the heart transplant population and how profiling antibody reactivities using this technology leads to new insights in both the pre- and post- transplant settings.

## Methods

### Patients and Sample Collection

Ethics approval (08-0732-T) for this project was obtained from the research ethics board at the University Health Network. Serum samples from patients who underwent heart transplantation from June 2003 to January 2011 were obtained from the UHN histocompatibility laboratory. For the study examining pre-transplant autoantibodies, there were 8 patients in the rejector group and 16 patients in the non-rejector group. Patients in the rejector group had at least two episodes of grade 2R rejection as defined by the International Society of Heart and Lung Transplantation (ISHLT) [18] in the first year after transplantation, whereas patients in the non-rejector group did not have any episodes of 2R rejection. In this classification, heart allograft biopsies are graded as 0R, 1R, 2R, or 3R based on multiple criteria including the presence of cellular infiltrates and myocyte necrosis. Healthy control samples were obtained from non-transplanted individuals from University Health Network and were age and sex matched to the rejector patients. For the study examining antibodies in AMR, there were 12 heart transplant recipients who were diagnosed with AMR and 19 recipients who did not have AMR (or cellular rejection). Non-AMR recipients were matched to AMR recipients to have a similar male to female sex ratio. AMR diagnosis was made according to echocardiographic evidence of decreased left ventricular ejection fraction (LVEF) and C4d or IgG deposition in the biopsy. The presence of donor-specific antibodies (DSA) in the serum was used to corroborate a diagnosis of AMR. Serum was taken from AMR patients at the time of AMR diagnosis and prior to plasmapheresis, whereas serum from non-AMR patients was matched for time post-transplant. cPRA (calculated panel-reactive antibody) was determined using the Canadian Blood Services web-based calculator using data from single antigen beads (One Lambda, Canoga Park, CA) on the Luminex platform (Luminex, Austin, TX).

### Antigen Library

Antigens were diluted to 0.2 mg/ml in PBS and stored in aliquots at -80°C. A complete list of all antigens arrayed is provided in the Supporting Information (Tables A-C in S1 File). Antigens were selected based on previous publications [19] with the inclusion of additional cardiovascular specific antigens. The arrays used to probe pre-transplant sera had 58 antigens and the arrays used to probe post-transplant sera had 64 antigens.

### Generation and Processing of Antigen Microarrays

Antigen microarrays specific for heart failure and heart transplantation were generated using previously published protocols [19, 20]. Antigens including proteins, peptides, and lysates were spotted in triplicate onto two-pad FAST nitrocellulose coated slides (Maine Manufacturing, Sanford, ME) using a VersArray Chipwriter Pro microarrayer (Virtek, Canada). Slides were arrayed at room temperature at a relative humidity of 55%. Solid Pins (Arrayit, Sunnyvale, CA) were used to generate features of approximately 500 microns in diameter. After the slides were completely dry, they were placed in FAST frames (Maine Manufacturing) and blocked overnight at 4°C in a blocking buffer (PBS, 5% FBS, 0.1% Tween). The following day, arrays were incubated with patient serum (1:150 diluted in blocking buffer) for one hour at 4°C. Slides were washed extensively with PBST (PBS with 0.1% Tween) and were probed with secondary antibodies and incubated for 45 minutes at 4°C. Slides were either probed with a mixture of secondary antibodies consisting of Cy3-labeled goat anti-human IgG (Jackson ImmunoResearch, West Grove, PA) at a dilution of 1:2000 and Cy5-labeled goat anti-human IgM (Jackson ImmunoResearch) at a dilution of 1:1000 or a single secondary antibody (Cy3-labeled goat anti-human IgG/IgM secondary antibody from Jackson ImmunoResearch) at a dilution of 1:2000. After additional washing, slides were dried by placing them in a rack and centrifuging (220 x g) for 5 minutes. In optimization experiments, human serum with known reactivity to ribosomal phosphoprotein P0 antigen (Immunovision, Springdale, AZ) was used as a positive control.

In studies comparing patients groups, arrays were probed and processed on the same day. The studies were then replicated on a different day using the identical protocol to ensure reproducibility of the results. Time-course data on select patients were generated by performing the protocol described above on samples collected retrospectively at several time points before and after transplant.

### ELISA for ribosomal phosphoprotein P0

Nunc Maxisorp plates (Ebioscience, San Diego, CA) were coated with ribosomal phosphoprotein P0 (Diarect, Germany) at a concentration of 10 μg/ml overnight at 4°C. Wells were incubated with human positive control serum with known reactivity to ribosomal phosphoprotein P0 (Immunovision, Springdale, AZ) at various dilutions in PBS containing 3% FBS and 0.05% Tween-20 followed by incubation with HRP-conjugated goat anti-human IgG secondary antibody (Jackson ImmunoResearch Laboratories) at a dilution of 1:5000. Tetramethylbenzidine substrate (Pierce) was added, and OD values were determined at 450 nm.

### Statistical Analysis

Analysis of antigen microarrays was performed as described in online protocols (http://robinsonlab.stanford.edu/microarrays/). Fluorescent intensities of features were quantified using an Axon 4200A microarray scanner (Molecular Devices, Sunnyvale, CA) and Genepix 6.1 software (Molecular Devices). For each feature, median fluorescent intensity minus local background (MFI-B) on the Cy3 (532nm) and Cy5 (635nm) channels was determined. The single averaged intensity for each antigen was calculated from the features arrayed in triplicate. Significance Analysis of Microarrays (SAM) [21] was used to determine significant changes in antigen reactivity between groups of patients with a false discovery rate of 5% (q-value < 0.05). Following SAM analysis, hierarchical clustering of antigens was performed with Cluster 3.0 [22] and heatmap displays were generated with Treeview 1.60 [22].

Step-wise discriminant analyses were performed to identify clinical variables (sex, age, diagnosis, cPRA class I, cPRA class II and VAD) and pre-transplant autoantibody levels (cardiac myosin, troponin I, ssDNA, ribosomal phosphoprotein P0 antigen, collagen I, collagen V, Hsp60 and Hsp27) that could be used in a function to classify patients as rejectors or non-rejectors. To minimize spurious entry of variables and to accommodate the small sample size (but obtain maximum potential for discernment), the maximum number of variables allowed to enter the functions was set at 3. Because some of the autoantibody variables displayed significant functional skewness, square root transformations were performed to reduce this property. Analyses were performed using SPSS 16.0 software (IBM, Armonk, NY).

Comparison of patient characteristics between groups was performed using a two-tailed unpaired T-test (for continuous variables with normal distribution) or alternatively, a non-parametric Mann-Whitney U test. Proportions of individuals with certain characteristics were compared between groups using a chi-square statistic. Correlation studies were performed by calculating Pearson correlation coefficients. A p-value of less than 0.05 was considered to be statistically significant. Analysis was performed using GraphPad Prism 6 software (GraphPad Software, La Jolla, CA).

## Results

### Generation of antigen microarrays

**Fig 1:**
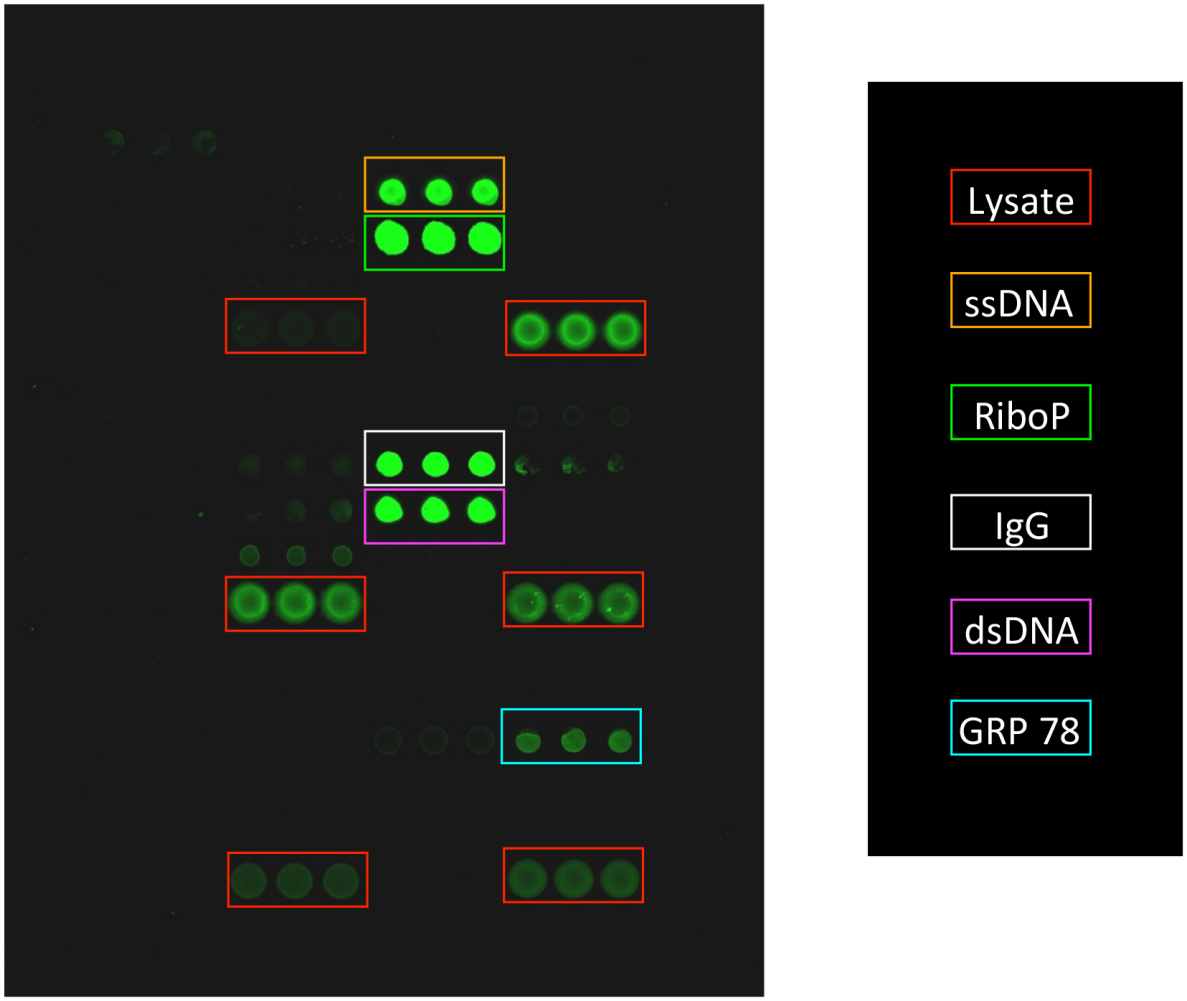
Scanned image of an antigen microarray probed with positive control serum. Array features were spotted in triplicate and then probed with serum with known reactivity to ribosomal phosphoprotein P0. A single Cy3-labelled secondary antibody that detects human IgG/IgM was used. Identity of antigens is indicated by the legend. IgG that is spotted onto the array is detected by the secondary antibody. Multiple reactivities are detected with the positive control serum including ribosomal phosphoprotein P0 (RiboP) (green box), single-stranded DNA (ssDNA) (orange box), double stranded DNA (dsDNA) (purple box) and the heat shock protein GRP78 (blue box). Red boxes indicate placement of six different cellular lysates. Array features are approximately 500 mm in diameter.

For our first generation antigen microarrays, we spotted 58 antigens in triplicate. These arrays included systemic lupus erythematosus (SLE) autoantigens, which have been previously validated, along with antigens that have been implicated in cardiovascular disease. We started by conducting proof of principle studies to determine whether our antigen microarray technique would be able to detect autoantibody reactivities known to be enriched in sera of patients with SLE. We therefore probed our arrays with a positive control serum with known reactivity to the SLE antigen ribosomal phosphoprotein P0 and then probed with a single Cy3-labeled secondary antibody that recognized both human IgG and IgM as previously described [13, 19]. Fig 1 shows an array probed with the positive control serum with known reactivity to ribosomal phosphoprotein P0. In addition to detecting autoantibodies to ribosomal phosphoprotein P0, the arrays detected additional reactivities (DNA and the heat shock protein Grp78), which are known to be present in this positive control serum [19].

**Fig 2:**
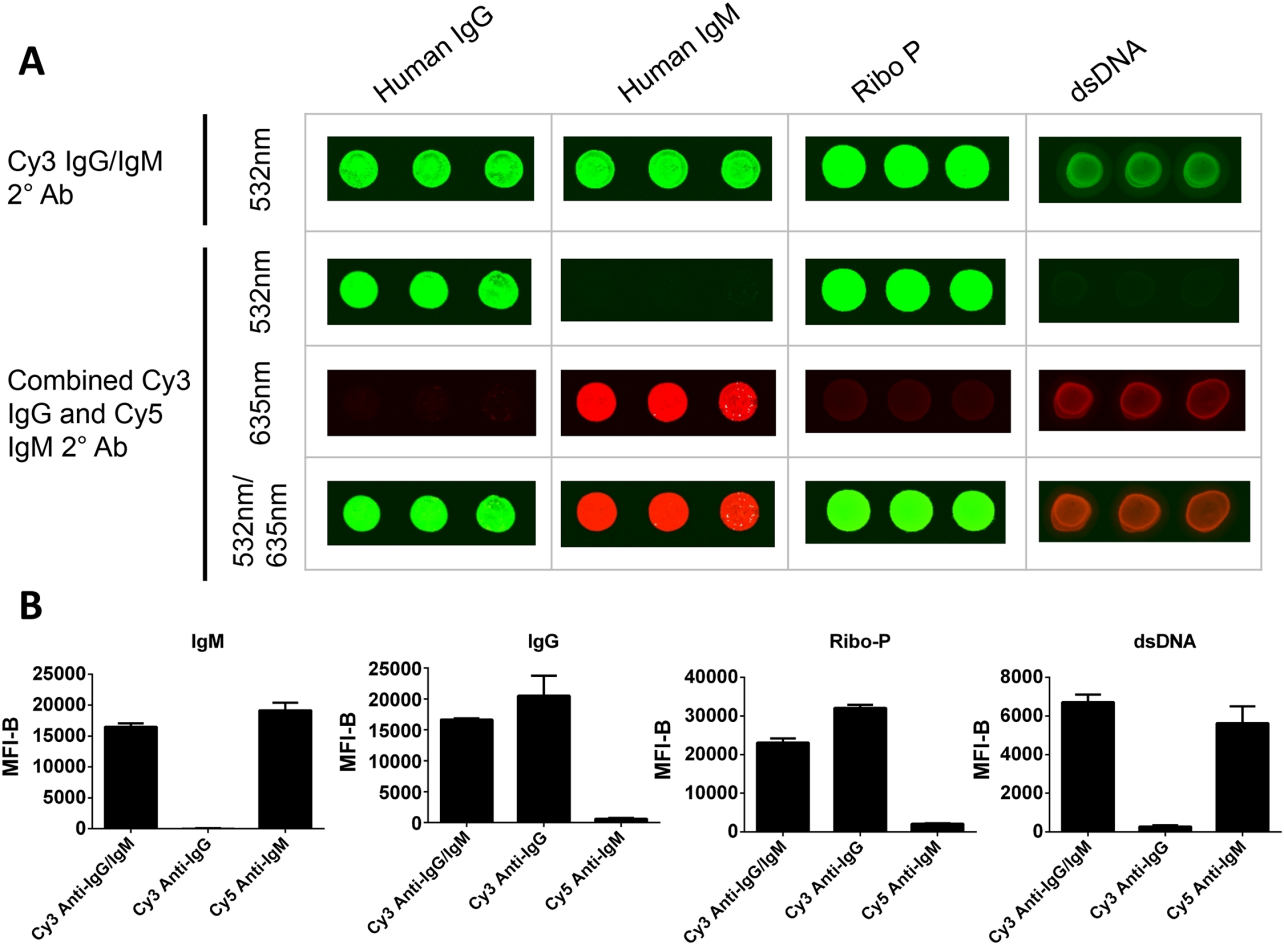
Generation of two-color antigen microarrays to detect IgM and IgG and comparison with single-color technique that detects IgG plus IgM antibody reactivities. (A) Human IgG, human IgM, ribosomal phosphoprotein P0, or dsDNA were spotted in triplicate onto nitrocellulose-coated slides. Slides were then probed with serum from a patient who was known to be reactive against ribosomal phosphoprotein P0 antigen. This was followed by probing with a Cy3-labeled secondary antibody that detected both IgM and IgG (top panel) or with two antibodies that specifically recognized IgM (Cy5-labeled) and IgG (Cy3-labeled) (bottom panels). Arrays were scanned and positive binding of the Cy3-labeled antibodies was detected at 532 nm while positive binding of the Cy5-labeled antibodies was detected at 635 nm as indicated. (B) Quantification of fluorescence detected from antigen microarrays at the two wavelengths. Graphs show mean ± SD median fluorescence intensity minus background (MFI-B) of the signal detected at the IgG, IgM, ribosomal phosphoprotein P0, and dsDNA spots at each wavelength. Each graph shows how reactivity detected by single secondary antibody (anti-IgG/IgM) can be separated into IgG and IgM reactivities using the antibody pair (anti-IgG and anti-IgM).

Once we were confident of the specificity of the arrays, we further refined the method by using two separate secondary antibodies that carried different fluorescent labels that could distinguish between human IgG and IgM. To demonstrate that the single reactivity detected by the anti-IgG/IgM secondary could be resolved into separate IgG and IgM reactivities using this secondary antibody pair, we probed the microarrays with serum that had known reactivities to the autoantigens ribosomal phosphoprotein P0 and double-stranded DNA (dsDNA) [19]. Slides were then probed either with the single Cy3-anti-IgG/IgM (top panel in Fig 2A) or the Cy3-anti-IgG and Cy5-anti-IgM mixture (bottom three panels in Fig 2A). We observed that the anti-IgG antibody detected only IgG spotted onto the array while the anti-IgM secondary detected only IgM (Fig 2A & 2B). We were also able to determine that ribosomal phosphoprotein P0 autoantibodies in this patient’s serum were predominantly of the IgG isotype, whereas the dsDNA autoantibodies were of the IgM isotype (Fig 2A & 2B). There was a small observable amount of reactivity against IgG in the Cy5 channel (anti-IgM channel), which may be explained by the presence of IgM autoantibodies against IgG (i.e., rheumatoid factor) in this patient’s serum.

To compare the sensitivity of our technique to the current gold standard of ELISA, we measured how much serum was required to detect ribosomal phosphoprotein P0 antibodies in this patient’s sera using both the ELISA and array methods. Similar to previous reports [12], we found that the antigen microarray had a 4-fold higher sensitivity for detection of ribosomal phosphoprotein P0 autoantibodies as compared to the traditional ELISA method (Table 1). Together, these results validate that our method has greater sensitivity to detect autoantibodies than the ELISA method.

**Table 1:** Enhanced sensitivity of antigen microarrays compared with ELISA to detect autoantibodies.

Ribo P Serum Dilution (1:N)	IgG Anti-Ribo P Antibodies	
	Microarray (MFI-B)	ELISA (OD)
1:250	64131	0.623
1:500	62598	0.535
1:1000	64349	0.422
1:2000	45765	0.291
1:4000	32019	0.169
1:8000	17611	0.103
1:16000	8313	0.062
1:32000	3983	0.041
1:64000	1878	0.025
1:128000	809	0.012
1:256000	462	ND
1:512000	202	ND

MFI-B, median fluorescence intensity minus background for microarray features; OD, optical density; RiboP, ribosomal phosphoprotein P0; ND, not detected

### Antigen Microarrays detect differences in pre-transplant serum between rejectors and non-rejectors

The ability to identify heart transplant recipients at higher risk of rejection is important in developing personalized immunosuppression and biopsy monitoring protocols. Since past studies have recognized that autoantibodies are correlated with rejection episodes, we decided to first pilot our antigen microarrays in the setting of heart transplantation. We investigated whether there was a correlation between specific pre-transplant autoantibodies and rejection following heart transplantation. Patients were designated as rejectors if they had ≥ 2 episodes of ISHLT grade 2R cellular rejection in the first year after transplantation, whereas non-rejectors had no episodes of 2R rejection. All heart transplant recipients received induction therapy as part of an institutional protocol. Clinical characteristics of rejector and non-rejector heart transplant recipients are shown in Table 2. As can be seen from the table, rejectors were younger in age, more likely to be female, and more likely to have a non-ischemic cardiomyopathy. Rejectors were also more likely to be sensitized pre-transplant with a higher cPRA for class I HLA antigens. Following heart transplantation, rejectors had more rejection episodes and had a higher biopsy score, calculated as described previously [23]. There was also a trend for rejectors to develop de-novo donor specific antibodies (DSA) (P = 0.052).

**Table 2:** Pre-Transplant Study: Comparison of rejector and non-rejector patient characteristics.

	Rejectors	Non-Rejectors	P value
Sample Size	8	16	
Male	25% (2/8)	56% (9/16)	P = 0.15
Age at Transplant (Years ± SD)	37.0 ± 13.0	51.3 ± 12.3	P = 0.02
Ischemic Cardiomyopathy	0%	38%	P = 0.05
Pre-Transplant cPRA Class I (% ± SD)	44 ± 37	5 ± 15	P = 0.02
Pre-Transplant cPRA Class II (% ± SD)	14 ± 34	1 ± 3	P = 0.31
Pre-Transplant VAD	25% (2/8)	12.5% (2/16)	P = 0.44
2R rejection episodes	2.4 ± 0.9	0	P<0.001
Total Rejection Score*	0.92 ± 0.29	0.28 ± 0.14	P<0.001
DSA within first year post-transplant	38% (3/8)	6% (1/16)	P = 0.05

DSA, donor specific antibody; VAD, ventricular assist device; cPRA, calculated panel-reactive antibody; SD, standard deviation.

* Total rejection score calculated as described previously[23].

**Fig 3:**
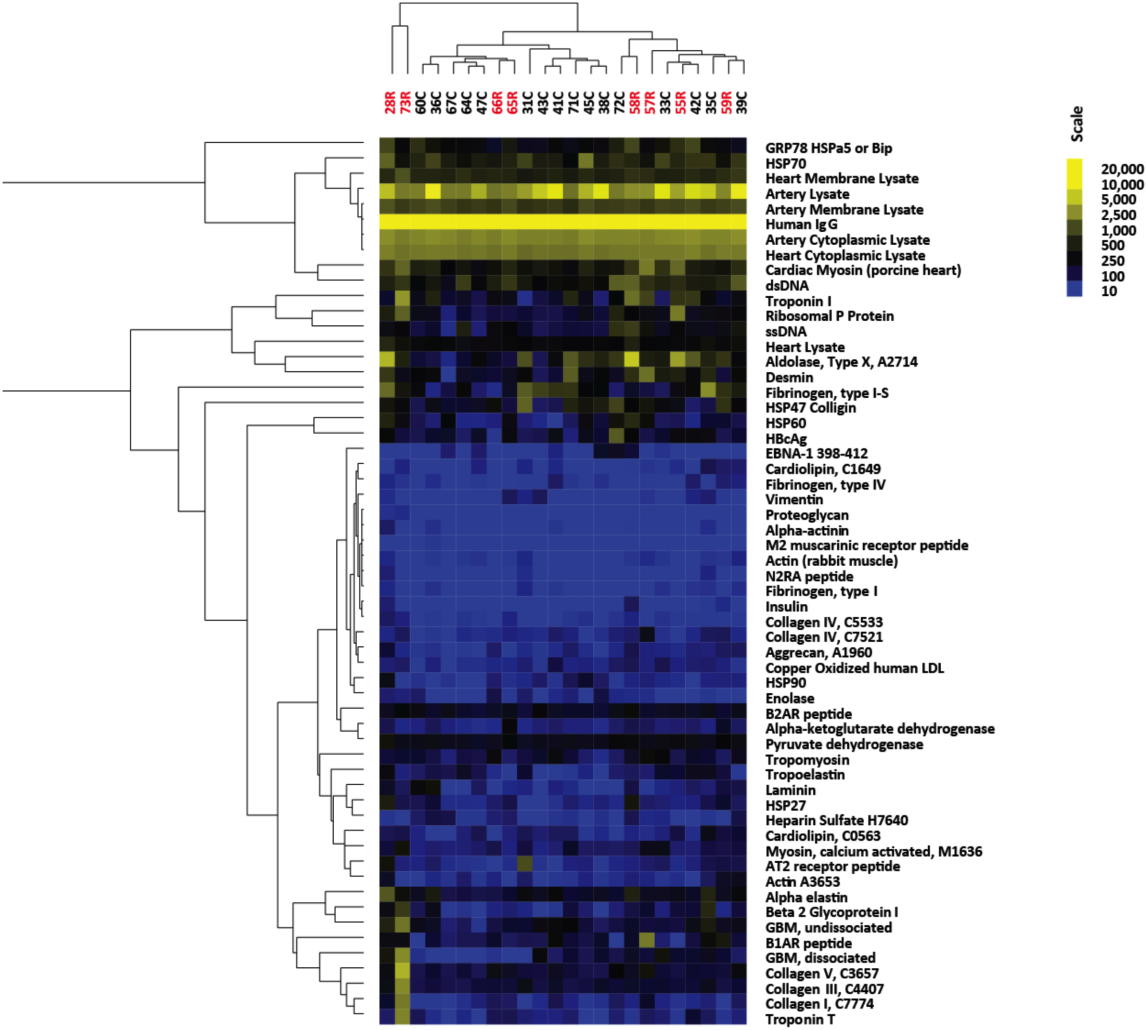
Heatmap of all autoantibody reactivities in pre-transplant serum from rejectors and non-rejectors. Yellow indicates higher reactivity, whereas blue indicates lower reactivity as shown in scale. Recipients who experienced rejection are in labeled in red and non-rejectors are labeled in black. Results are representative of two independent array experiments.

**Fig 4:**
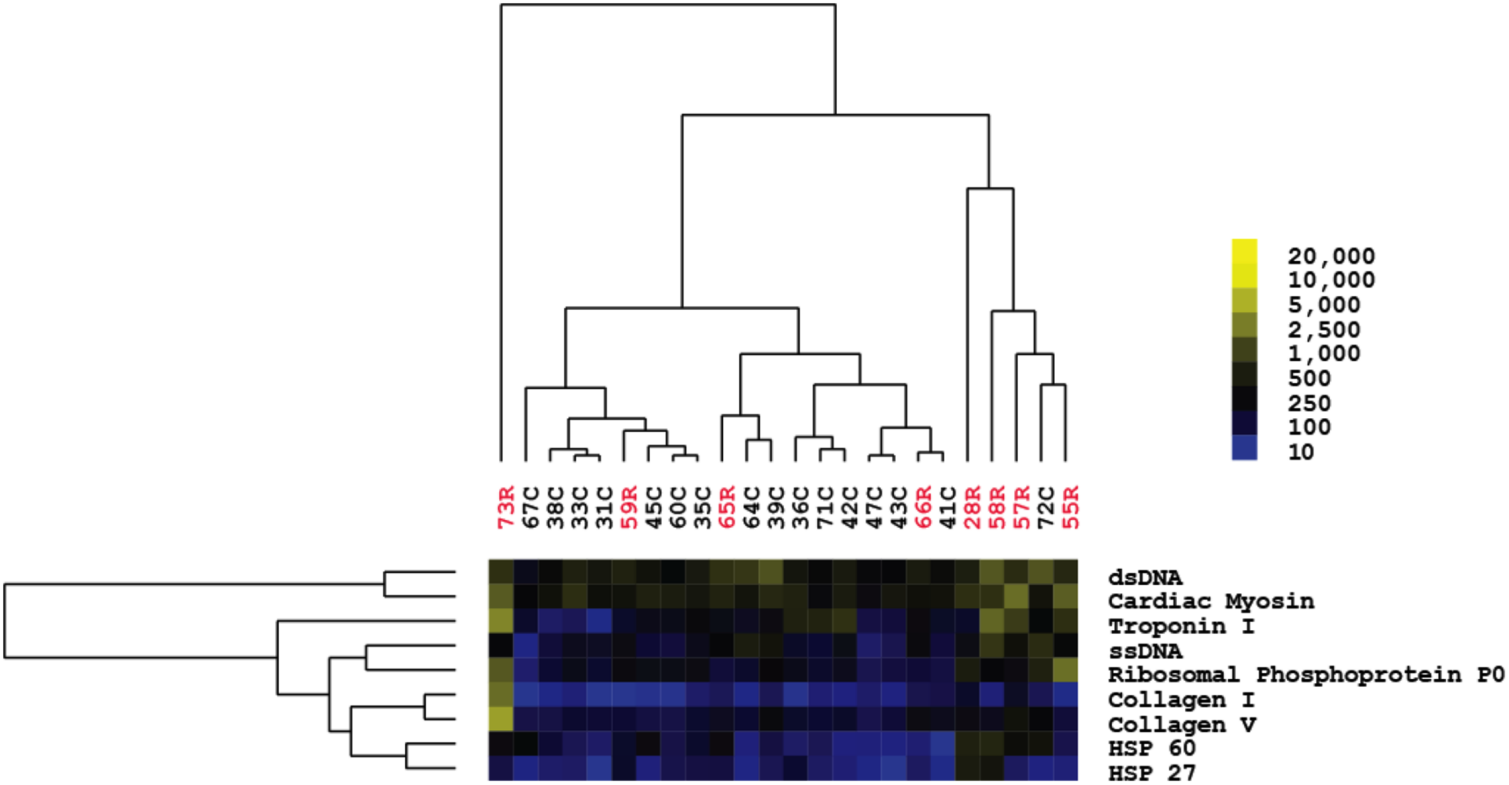
Heatmap showing reactivities that are higher in pre-transplant serum from rejectors compared with non-rejectors. Rejectors are indicated in red. Significant differences between rejectors and non-rejectors were detected with the SAM algorithm with q value < 0.05. Four of the rejectors are grouped together at the right of the heatmap and the rejector with the highest reactivities is in a separate group at the left. Scale shows reactivity from low (blue) to high (yellow). Results are representative of two independent array experiments. dsDNA, double-stranded DNA; Hsp60, heat shock protein 60; Hsp27, heat shock protein 27; ssDNA, single-stranded DNA.

Antigen microarrays were performed on pre-transplant sera from patients who went on to develop rejection (rejectors) or were free from rejection (non-rejectors) using a single secondary antibody that detects both IgG and IgM. The arrays, which had 58 antigens spotted in triplicate, detected a wide range of reactivity to multiple antigens including cardiac antigens such as cardiac myosin, troponin I, and the beta 1 adrenergic receptor (Fig 3). In order to determine significant differences in antigen reactivity between the rejector and non-rejector groups, SAM analysis was performed, which revealed eight antigen reactivities to be significantly elevated in the rejector group versus the non-rejector group (Fig 4 and Table D in S1 File). No antigen reactivities were decreased in the rejector group compared with the non-rejector group. Amongst these distinguishing autoantibodies, anti-cardiac myosin antibodies were elevated in the rejector group. This is consistent with prior studies that demonstrated that the presence of pre-transplant antibodies to myosin is correlated with increased rates of rejection after heart transplantation [4]. SAM also identified reactivity to troponin I, single-stranded DNA, ribosomal phosphoprotein P0, heat shock protein 27 (Hsp27), and heat shock protein 60 (Hsp60), collagen I, and collagen V to be elevated in the rejector group compared with the non-rejector group. However, for collagen I and V, the changes appeared to be primarily driven by one patient (73R).

Antigen microarrays were also performed to compare antibody profile between patients in the rejector group and healthy controls in order to determine if the identified significant reactivities in the rejector group were also higher than in healthy controls. Healthy controls were sex and age matched to the patients in the rejector group. Not surprisingly antibody reactivities to heart-associated proteins myosin, troponin I, and aldolase were significantly upregulated in the rejector group as compared with the healthy controls by SAM analysis (Table E in S1 File). The heat-shock protein Hsp60 was also higher in the rejectors, consistent with the possibility of increased heart damage in the rejector group.

### Using autoantibody levels to predict a rejector phenotype after heart transplantation

In current clinical practice, clinical information such as a patient’s age, sex, and levels of pre-formed anti-HLA antibodies as measured by cPRA are used to predict the probability of a rejection phenotype post-transplant. For example, younger female patients typically exhibit stronger adaptive immune responses and are reported to be more likely to reject their organs [24]. To determine whether the autoantibody reactivities that we measured added any predictive power to current clinical information, we performed a series of step-wise discriminant analyses (max steps = 3) with rejection (yes or no) as the dichotomous variable and age, sex (male or female), diagnosis (ischemic or non-ischemic), history of VAD (yes or no) and MFIs of autoantibodies identified by SAM as predictive variables. In the first analysis, we allowed the current clinical information variables (age, sex, diagnosis, VAD, cPRA class I, cPRA class II) to enter. The resulting discriminant function was composed of two variables (age and cPRA class I) and explained 50% of the variance in rejection and correctly classified 88% (14/16) of the non-rejectors and 88% (7/8) of the rejectors (Table 3). We then repeated the discriminant analyses except allowed all variables to enter including the autoantibodies. This analysis was done using either the raw MFI values for the autoantibodies or square root (sqrt) transformations of these values to reduce the skewness of these data. When all of the variables were allowed to enter including the sqrt of autoantibody MFIs, the discriminant function was composed of three variables (cPRA class I, sqrt Hsp27 autoantibody levels, and sqrt ribosomal phosphoprotein P0 autoantibody levels) and explained 72% of the variance and correctly classified all (100%) of the non-rejectors and 88% (7/8) of the rejectors (Table 3). Similar results were obtained using raw autoantibody values (Table 3). Together, these results indicated that information about Hsp27 and ribosomal phosphoprotein P0 autoantibody levels provided additional and unique information from cPRA class I in helping to predict the probability of a rejector phenotype.

**Table 3:** Step-wise discriminant analyses of pre-transplant variables that predict rejection outcome (Yes/No) (maximum 3 steps).

**Analysis 1: Clinical variables allowed to enter (age, sex, diagnosis, cPRA class I, cPRA class II, VAD)**	
Variables	Canonical Discriminant Function Coefficients
cPRA Class I	0.034
Age	-0.047
(Constant)	1.590
Correctly classified Cases (%)	
Non-Rejectors	88%
Rejectors	88%
Chi-square (df = 2) (p value)	14.26 (p = 0.001)
Canonical Correlation	0.702
Centroids	
Non-rejectors	-0.67
Rejectors	1.33
**Analysis 2: All variables allowed to enter (autoantibody MFI-B were square root transformed)**	
Variables	Canonical Discriminant Function Coefficients
cPRA Class I	0.039
Sqrt Hsp27	0.210
Sqrt Ribosomal phosphoprotein P0	0.069
(Constant)	-3.364
Correctly classified Cases (%)	
Non-Rejectors	100%
Rejectors	88%
Chi-square (df = 3) (p value)	26.26 (p<0.001)
Canonical Correlation	0.85
Centroids	
Non-rejectors	-1.09
Rejectors	2.18
**Analysis 3: All variables allowed to enter (autoantibody MFI-B were not transformed)**	
Variables	Canonical Discriminant Function Coefficients
cPRA Class I	0.41
Hsp27	0.009
Ribosomal phosphoprotein P0	0.002
(Constant)	-1.919
Correctly classified Cases (%)	
Non-Rejectors	93%
Rejectors	88%
Chi-square (df = 3) (p value)	27.23 (p<0.001)
Canonical Correlation	0.86
Centroids	
Non-rejectors	-1.128
Rejectors	2.255
**Analysis 4: All variables allowed to enter (using data from first 4 rejectors and first 8 non-rejectors)**	
Variables	Canonical Discriminant Function Coefficients
cPRA Class I	0.093
Sqrt Hsp27	0.287
Sqrt Ribosomal phosphoprotein P0	0.249
(Constant)	-7.536
Correctly classified Cases (%)	
Non-Rejectors	100%
Rejectors	100%
Chi-square (df = 3) (p value)	23.65 (p<0.001)
Canonical Correlation	0.97
Centroids	
Non-rejectors	-2.51
Rejectors	5.03

cPRA, calculated panel-reactive antibody; df, degrees of freedom; MBI-B, median fluorescence intensity minus background; VAD, ventricular assist device

In order to discern the reliability of the aggregate of cPRA class I, ribosomal phosphoprotein P0 autoantibody levels and Hsp27 autoantibody levels to discriminate rejectors and non-rejectors, we also conducted a split analysis, where we obtained the discriminant function for the first 4 rejectors and the first 8 non-rejectors in our subject list. The canonical correlation was 0.97 (chi-squared = 23.65) and classified 100% of the cases (Table 3). When this function was applied to the remaining patients (4 rejectors, 8 non-rejectors) whose scores had not been involved obtaining the function, chi-squared (4.69, p = 0.03) analysis indicated 83% accurate classification with only one patient from each group being misclassified. In spite of the small size of the data set, these data suggest a generalizability of these three variables to predict rejection following transplantation.

### Antigen Microarray detect differences in post-transplant serum between AMR and non-AMR patients

To identify reactivities that distinguished heart-transplant patients with AMR in a retrospective study, we probed these arrays with serum samples taken from 12 heart transplant patients who were diagnosed with AMR and 19 non-AMR heart transplant patients. AMR patient serum was sampled at the time of AMR diagnosis, whereas the serum from non-AMR patients was taken at a similar span of time post-transplant. The clinical characteristics of these patients are shown in Table 4. As expected, AMR patients had a lower mean LVEF than non-AMR patients. AMR patients were significantly younger and had higher levels of pre-transplant class I and II cPRA than non-AMR patients. There were no statistically significant differences between the two groups in terms of sex ratio, heart failure etiology, pre-transplant ventricular assist device (VAD), total cellular rejection score [23], time of sample collection post-transplant, or immunosuppression.

**Table 4:** Post-Transplant Study: Comparison of AMR and non-AMR patient characteristics.

	AMR	Non-AMR	P value
Sample Size	12	19	
Male	75% (9/12)	68% (13/19)	1.00
Age at Transplant (Years ± SD)	40.2 ± 12.7	52.4 ± 10.4	0.007
Ischemic Cardiomyopathy	25% (4/12)	42% (8/19)	0.71
Pre-Transplant cPRA Class I (% ± SD)	34 ± 34	3 ± 8	0.002
Pre-Transplant cPRA Class II (% ± SD)	28 ± 38	2 ± 9	0.008
Pre-Transplant VAD	42% (5/12)	11% (2/19)	0.08
Total Rejection Score*	0.33 ± 0.23	0.29 ± 0.14	0.48
Time of Sample Post-Transplant (Years ± SD)	1.92 ± 1.94	1.71 ± 0.78	0.73
LVEF (% ± SD)	43 ± 12%	> 60%	0.003
C4d positive biopsy^†^	58% (7/12)	11% (2/19)	0.012
DSA	75% (9/12)	0%	0.0001
Prednisone	83% (10/12)	84% (16/19)	1.00
MMF	92% (11/12)	68% (13/16)	0.20
mTOR inhibitor	33% (4/12)	26% (5/19)	0.69
Cyclosporine	58% (7/12)	53% (10/19)	1.00
Tacrolimus	42% (5/12)	47% (9/19)	1.00

DSA, donor specific antibody; VAD, ventricular assist device; cPRA, calculated panel-reactive antibody; LVEF, left ventricular ejection fraction; MMF, mycophenolate mofetil; SD, standard deviation.

* Total rejection score calculated as described previously [23].

^†^ Two non-rejectors had transient C4d staining on the biopsy preceding the serum sample. C4d staining was not observed on a subsequent biopsy in these patients.

**Fig 5:**
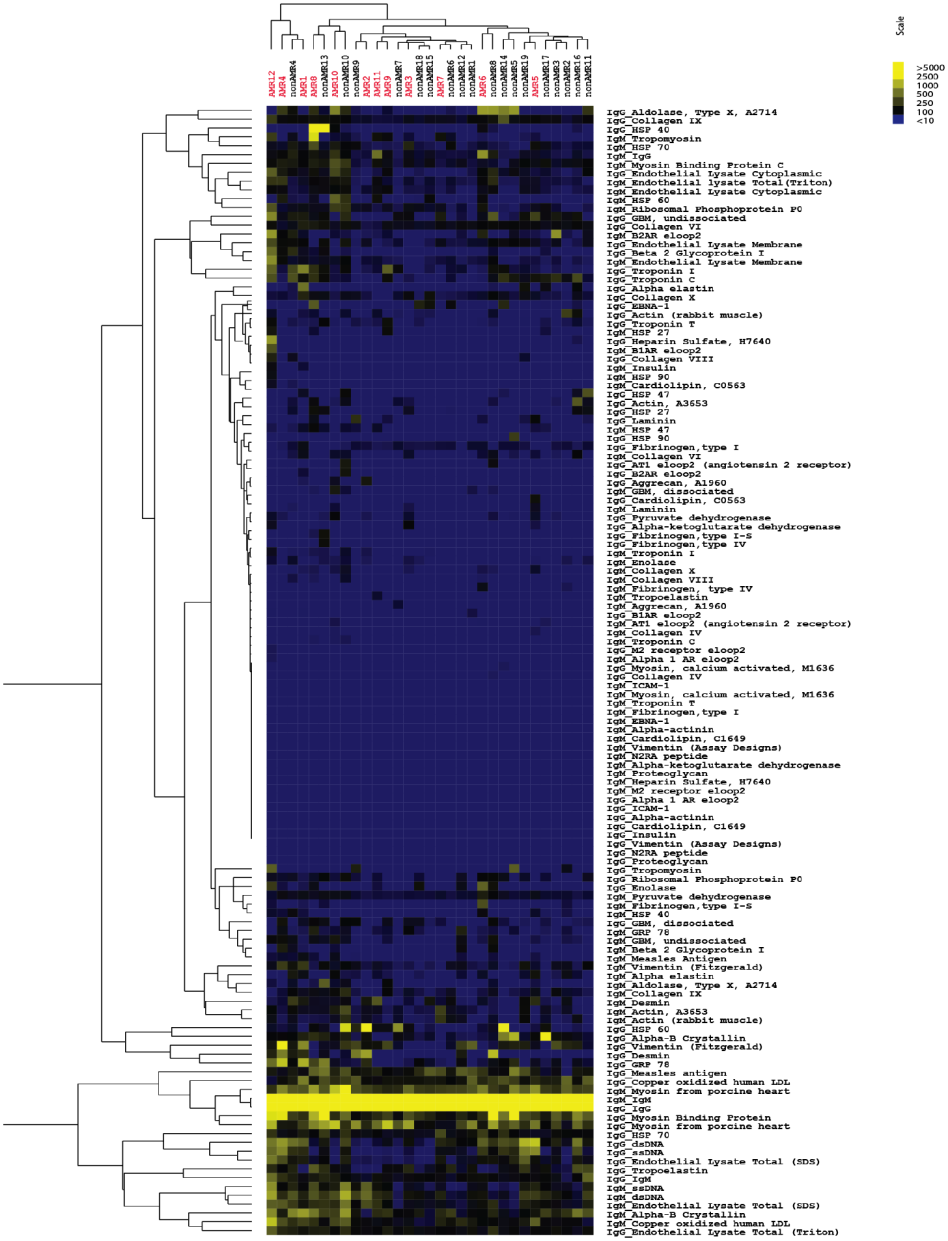
Heatmap of all non-HLA reactivities in post-transplant serum of AMR and non-AMR patients. Yellow indicates higher reactivity, whereas blue indicates lower reactivity as shown in scale. Recipients with AMR are in labeled in red and are numbered 1–12. Non-AMR patients are labeled in black and numbered 1–19. Results are representative of two independent array experiments.

**Fig 6:**
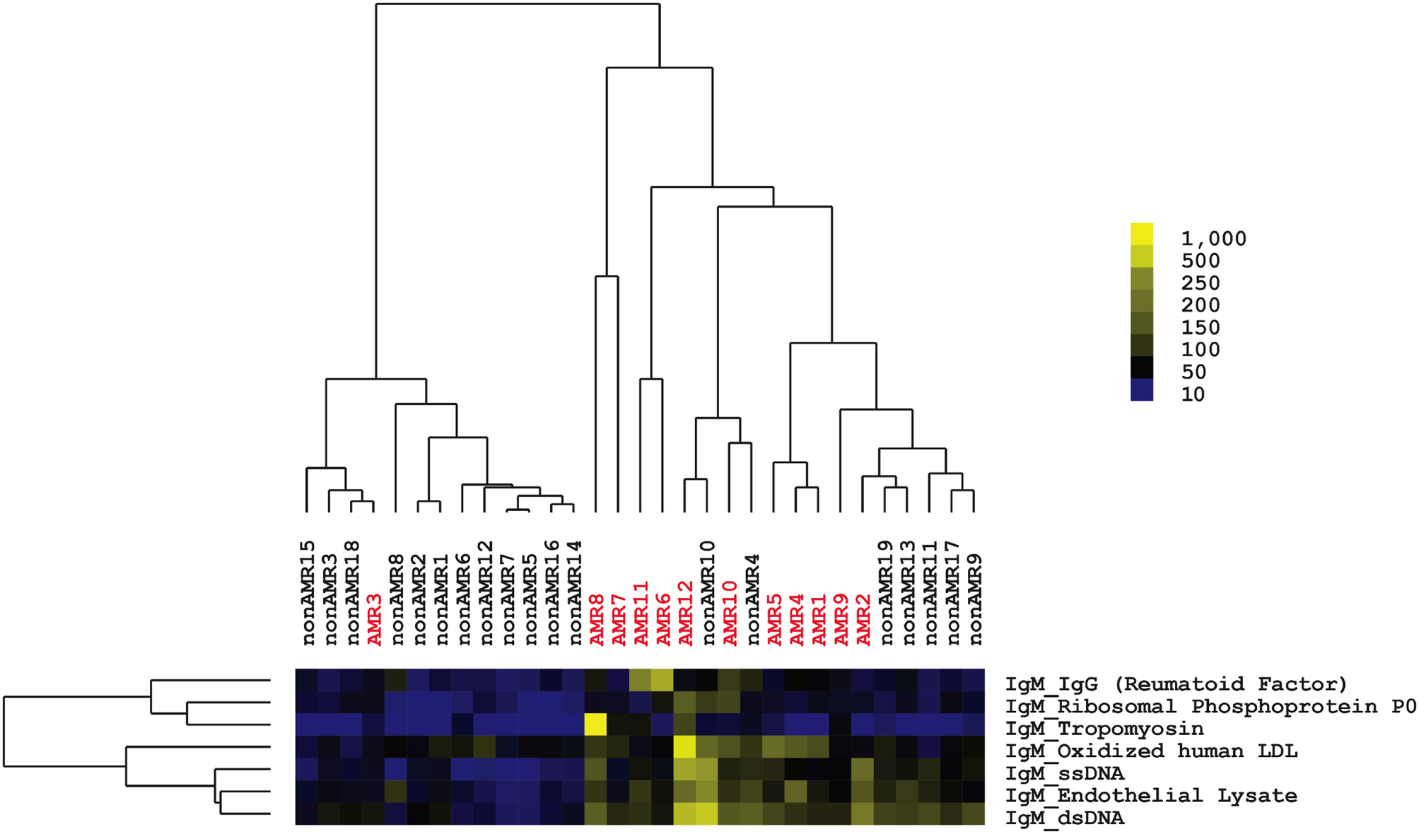
Heatmap of non-HLA reactivities that are significantly higher in post-transplant serum of AMR patients compared with non-AMR patients. Significance analysis of microarrays identified seven antigen reactivities that are higher in recipients with AMR compared with non-rejectors. Yellow squares in the heatmap indicate a higher reactivity, whereas blue squares indicate a lower reactivity as shown in scale. Recipients with AMR are labeled in red and are numbered 1–12. Non-rejectors are labeled in black and numbered 1–19. Results are representative of two independent array experiments. ssDNA, single-stranded DNA; dsDNA, double-stranded DNA.

Microarrays were probed with patient serum to determine antibody reactivity to all 64 antigens on the arrays. The pair of Cy3-anti-IgG and Cy5-anti-IgM secondary antibodies was used to distinguish non-HLA antibody isotype. A heatmap with all patient reactivities is shown in Fig 5. SAM and hierarchal clustering analysis was then applied to identify antigens that exhibited significant differences in reactivity between groups. These analyses identified seven non-HLA antibodies that could cluster patients in the AMR and non-AMR groups based on similar reactivity patterns (Fig 6). These seven non-HLA antibodies were all at significantly higher levels (1.9 to 2.7 fold higher) in the AMR than the non-AMR group and were all of IgM isotype (Table F in S1 File). Patients with AMR patients exhibited higher levels of IgM antibodies reactive to double-stranded DNA (dsDNA), single-stranded DNA (ssDNA), ribosomal phosphoprotein P0, oxidized human LDL, endothelial cell lysates, and tropomyosin compared with the non-AMR patients. Two patients (AMR6 and AMR11) also exhibited high levels of IgM antibodies that were reactive to IgG, indicating the presence of rheumatoid factor. There was also a trend for patients with AMR to have higher levels of IgG reactivity to cardiac myosin, which has been previously observed in patients with AMR [11]. In order to test the reliability of these results, we also randomly divided the cohort into two groups and found that similar patterns of IgM anti-HLA antibodies were elevated in the patients with AMR vs. non-AMR in both groups (Table G in S1 File).

### Time-course analyses of non-HLA IgM antibodies in two AMR patients

**Fig 7:**
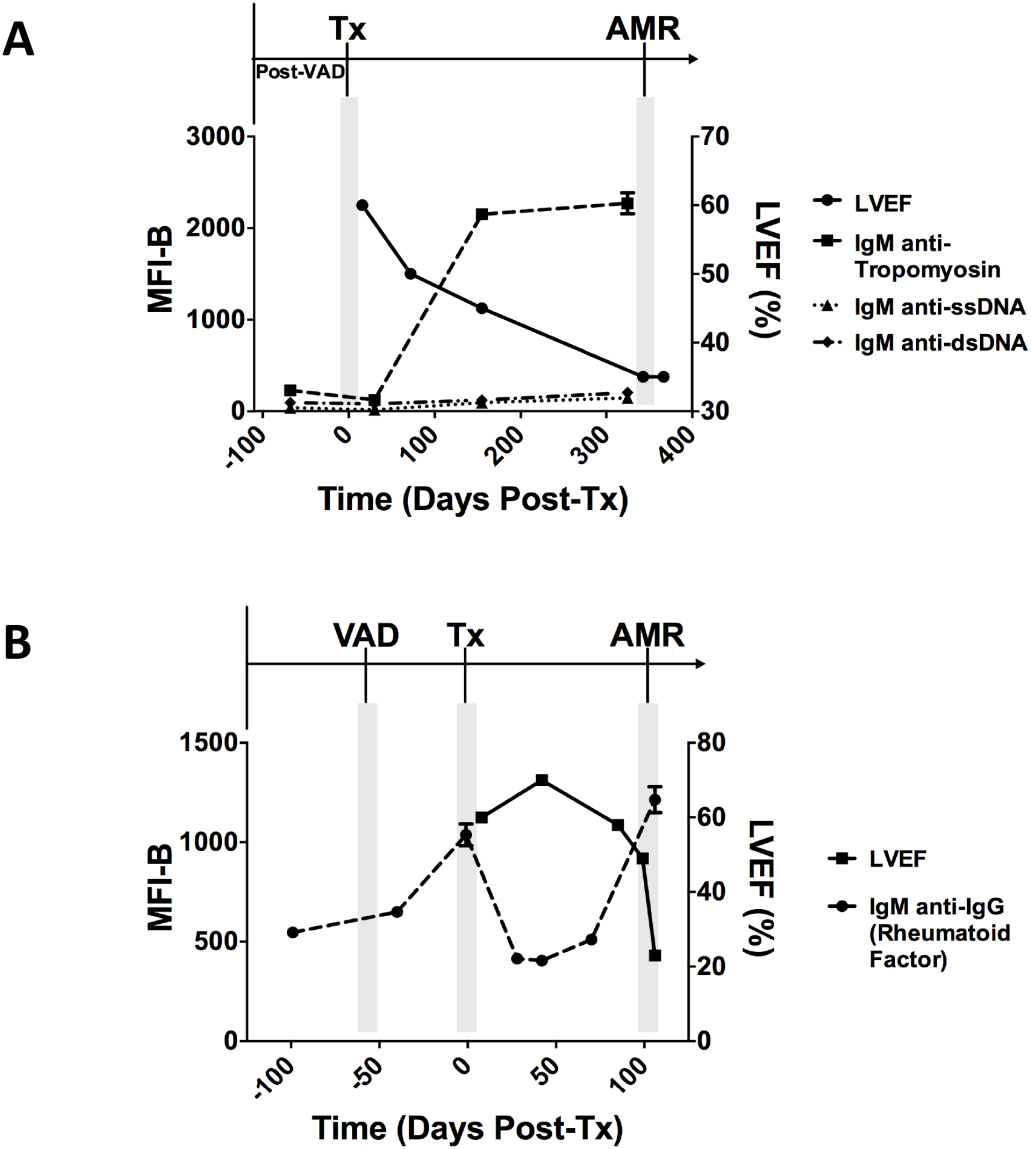
Time course of ejection fraction and non-HLA antibodies in two patients with AMR. The solid line plots left ventricular ejection fraction and the dashed lines plot median fluorescence intensity minus background (MFI-B) of various antigens over time. Timing of VAD placement, heart transplant (Tx), and diagnosis of AMR are indicated above the graphs. Day of heart transplant is designated as Day 0. Graph shows mean ± SD for array features. (A) Patient AMR8. This patient developed IgM anti-tropomyosin and to a lesser extent IgM anti-DNA antibodies following transplant, which coincided with a drop in graft function. (B) Patient AMR6. This patient developed IgM anti-IgG antibodies (rheumatoid factor) following VAD placement, which decreased following heart transplant. A severe drop in graft function coincided with reappearance of rheumatoid factor. dsDNA, double-stranded DNA; LVEF, left ventricular ejection fraction; MFI-B, median fluorescence intensity minus background; ssDNA, single-stranded DNA; VAD, ventricular assist device.

To investigate the chronological relationship between non-HLA antibody appearance in serum and overt graft dysfunction, we profiled antibody reactivities in two patients over time. We chose the two patients based on sample availability and because they had remained negative for DSA throughout the period of observation. Fig 7 shows time-course of fluorescence of distinct antigens that showed high reactivity in patients AMR6 and AMR8, superimposed onto LVEF. Patient AMR8 was distinguished by high levels of IgM anti-tropomyosin and lower-levels of IgM anti-dsDNA and IgM anti-ssDNA (Fig 7A). All three of these antibodies were present at low levels at one month post-transplant. Five months post-transplant, the levels of IgM anti-tropomyosin markedly increased, concurrent with a modest decrease in LVEF and at a time when the patient was still negative for C4d staining on the endomyocardial biopsy. The patient was not diagnosed with AMR until one year post-transplant, at a time when graft function further declined and C4d-staining became positive.

The second patient profiled, AMR6, had one episode of 2R cellular rejection in the early post-transplant period followed by multiple episodes of 1R rejection. During this period, the patient had preserved LVEF and low rheumatoid factor reactivity. At the time of diagnosis of AMR, the patient experienced a significant decrease in LVEF and heart biopsies demonstrated positive IgG staining but negative C4d staining. Clinical graft dysfunction was accompanied by a three-fold increase in the levels of rheumatoid factor (Fig 7B). There was also a small increase in rheumatoid factor prior to the drop in graft function. Interestingly, our time-course data for this patient revealed an increase in rheumatoid factor after VAD implantation, suggesting that the device may have initiated an inflammatory process. Together, these cases highlight the possible clinical utility of measuring non-HLA antibodies over time to monitor heart transplant recipients.

## Discussion

Autoantibodies are increasingly being recognized as important components of the host immune response in patients with heart failure and in heart transplant recipients. Here, we describe the generation of custom antigen microarrays to profile autoantibodies in the pre- and post-heart transplant settings. We have further refined previously described technologies to simultaneously quantify IgG and IgM reactivities for a variety of antigens including protein, peptides, and cell lysates. In a retrospective study on pre-transplant serum, we found that several antibody reactivities correlated with increased rates of cellular rejection following heart transplantation. In another study, we identified seven IgM reactivities that were higher in the heart transplant recipients with AMR compared with control recipients. Time course studies in two patients with AMR demonstrated that specific IgM non-HLA antibodies preceded the diagnosis of AMR and thus may signal early graft damage prior to overt graft dysfunction.

Antigen microarrays offer several advantages over traditional ELISA assays in detecting and quantifying autoantibodies. First, antigen reactivities are profiled in multiplex fashion on the arrays, allowing for many reactivities to be defined simultaneously [12]. Second, the arrays are fully customizable and new antigens can be easily added. In this report, we profiled 64 antigens; however, our current array platform has the capacity for 162 antigens printed in duplicate so there is ample space to expand the arrays to include additional antigens. Third, the arrays are cost-effective as minimal amounts of antigen (less than 10 nL) are spotted onto the slides. Thus, the same amount of recombinant protein that is needed to coat one ELISA plate with 96 wells could be used to spot over 10,000 antigen microarrays. Patient samples are also used sparingly as one array is typically probed with only 5 microliters of patient sera/plasma diluted in buffer. Finally, as previously observed, we have found that arrays are at least 4 fold more sensitive than conventional ELISAs at detecting individual antibody reactivities. Because of these characteristics, antigen microarrays are ideally suited for autoantibody screening in research and clinical practice.

In the first set of experiments, we retrospectively profiled sera from heart failure patients who underwent heart transplantation and were subsequently divided into rejectors and non-rejectors. We identified eight antigen reactivities in pre-transplant sera that were upregulated in rejectors compared with the non-rejectors. Antibodies to cardiac myosin, which have been previously identified as a risk factor for rejection [4], were found to be higher in rejectors in this study. Antibodies to cardiac myosin have been reported in patients with heart failure and may have a direct pathologic role in cardiac dysfunction [25]. Studies in mice have shown that passive transfer of anti-cardiac myosin antibodies can lead to myocarditis in susceptible strains [26]. We also identified that autoantibodies to troponin I were elevated in rejectors compared with both non-rejectors and healthy controls. Autoantibodies to troponin I have previously been identified in patients with heart failure, but their association with allograft rejection has not been reported. Antibodies to troponin I have been shown to lead to cardiomyopathy and heart failure in programmed death (PD)-1 knockout mice [27]. In these mice, autoantibodies to troponin I stained the surface of murine cardiac myocytes and may have led to myocyte damage by chronically activating calcium currents. These results suggest that antibodies to troponin I may also lead to allograft destruction, thereby promoting allograft rejection.

The other antibodies that were upregulated in pre-transplant serum from the rejectors were against ribosomal phosphoprotein P0, single-stranded DNA, Hsp27, Hsp60, collagen I, and collagen V. It is unclear if these antibodies are pathologic or if they are formed secondary to myocyte damage in the setting of heart failure. During cellular stress, heat shock proteins have been shown to be upregulated and may appear in the systemic circulation [28]. Antibodies may then develop against the heat shock proteins. Antibodies to Hsp27, for example, are elevated in patients with acute coronary syndromes and may be markers of cardiac myocyte necrosis [29]. Antibodies to Hsp60 have been shown to be elevated in patients with atherosclerotic heart disease and may induce endothelial dysfunction [30]. Antibodies to Hsp60 were also found to be elevated in patients with non-ischemic cardiomyopathies and levels of anti-Hsp60 antibodies correlated with the severity of left ventricular dysfunction [28].

Once we identified which autoantibodies were elevated in the rejector group, we performed stepwise discriminant analyses to determine if these autoantibodies added information to current clinical measures for predicting rejection outcomes. First, we examined the predictive power of currently used clinical measures and found that cPRA class I followed by age were important clinical variables in identifying patients who are likely to develop rejection following heart transplantation. Previous studies have demonstrated that a high PRA correlates with increased rates of rejection following heart transplantation [31]. Furthermore, the finding that age negatively correlates with rejector phenotype has been described previously and may relate to the development of more robust adaptive immune responses in younger patients [24, 32]. When we performed the discriminant analysis allowing both clinical variables and information on autoantibodies to enter, cPRA class I again loaded first followed by Hsp27 and ribosomal phosphoprotein P0 autoantibody levels. Importantly, information on the autoantibodies added to the clinical variables and improved the classification of patients into rejector and non-rejector groups. The finding that myosin and troponin I autoantibody levels did not enter the function do not discount a role for these autoantibodies in the rejection process. The reason that they did not enter is because all variance related to cPRA class I would have been removed after this variable entered the function, and we found in correlation studies that levels of autoantibodies to cardiac myosin (r = 0.501, p = 0.013) and troponin I (r = 0.548, p = 0.006) were positively correlated with cPRA class I. Future studies will further investigate the connection between anti-HLA antibodies and autoantibodies in patients with heart failure and explore the role of autoantibodies in promoting allograft rejection.

Measuring autoantibodies is not only important for predicting rates of rejection post-transplant, but also in identifying heart failure patients who may be candidates for treatment that would delay or obviate the need for transplantation. For example, therapies are being currently developed to neutralize pathogenic autoantibodies that bind to and activate the beta 1 adrenergic receptor in heart failure patients [33]. There are also reports that immunoadsorption therapy, which removes antibodies, can enhance heart function in patients with dilated cardiomyopathy [34]. Our antigen microarrays could be used as a screening test to select heart failure patients with evidence of autoimmunity who may benefit from these types of therapies. We are currently working to define the autoantibody repertoire (including levels as assessed by MFI) in larger cohorts of heart failure patients.

We also applied antigen microarrays to profile non-HLA antibodies in heart transplant recipients with AMR. In this study, we utilized two-color arrays to identify IgG and IgM reactivities separately. One of the important findings of this study was that heart transplant recipients with AMR developed a distinct profile of IgM antibodies against non-HLA antigens compared with recipients who were AMR-free. The finding that these antibodies were of the IgM isotype suggest that they are likely related to damage within the graft early during the rejection process. Indeed, IgM or natural antibodies have been described in the literature to play a key role in the clearance of apoptotic cell debris [35]. These antibodies bind with low affinity to both pathogenic and endogenous antigens, including epitopes revealed by apoptosis such as phosphorylcholine, oxidation products such as oxidized LDL, and intracellular antigens released during cell death such as dsDNA [36]. By recruiting complement component C1q and mannose-binding lectin, they signal the clearance of apoptotic components to prevent inflammation and autoimmunity [36]. Indeed, in support of this concept, intravenous infusion of apoptotic thymocytes into mice was shown to increase circulating IgM antibodies to oxidized LDL, phosphorylcholine and nucleic acid antigens [37]. Presumably, endothelial and myocyte injury in AMR can expose antigens (e.g., endothelial cell lysate antigens and tropomyosin) to immune surveillance, leading to feedback upregulation of IgM autoantibodies to aid in the phagocytosis of cellular debris. Thus, serial profiling of autoantibodies may be a new way to monitor graft damage in AMR. An FDA approved assay (Allomap) is currently available to identify heart transplant recipients with cell-mediated rejection [38]. In this assay, a score is calculated using gene expression from peripheral blood mononuclear cells that can be used to predict cell-mediated rejection. Unlike cell-mediated rejection, there is currently no commercially available biomarker assay for AMR in heart transplantation. Although the appearance of donor-specific anti-HLA antibodies in blood may be suggestive of a diagnosis of AMR, these antibodies are not by themselves diagnostic [7]. The presence of non-HLA antibodies as detected by antigen microarrays may therefore provide additional information in monitoring for AMR in a noninvasive fashion.

Though the seven significant antigens were able to cluster patients into AMR and non-AMR groups, some antibody reactivities were more predominant in some AMR patients than in others (e.g., IgM anti-tropomyosin in AMR8). Such a high degree of variability amongst the AMR patients suggests that AMR is characterized by a broad autoantibody “profile” and that multi-antigen panels, rather than single antibody testing, may be required for monitoring non-HLA antibodies. This individuality in autoantibody profile has been seen before among kidney allograft recipients with chronic humoral rejection [17]. Antigen microarrays have the capability to screen multiple autoantibodies simultaneously and due to their high sensitivity, can resolve differences at very low antibody concentrations. These attributes make this technique ideal for studying system-based antibody responses in the allogeneic transplant setting.

In conclusion, we have developed custom antigen microarrays to profile autoantibodies in heart failure and heart transplantation. In retrospective pre- and post-transplant studies, we were able to highlight the value of the arrays in detecting new antibody reactivities in these patients. We are in the process of validating these studies using patient samples from additional transplant centers. Given the increasing understanding of the immune system in cardiovascular disease, we expect that these arrays will be useful in detecting autoantibodies in many cardiovascular disorders.

## Supporting Information

S1 File Table A, Antigen List for First Study (rejector vs. non-rejector). Table B, Antigen List for Second Study (AMR vs. non-AMR). Table C, Sequence of G-protein Coupled Receptor Peptides. Table D, Fold change and q-value for autoantibodies upregulated in pre-transplant sera from rejectors compared with non-rejectors. Table E, Fold change and q-value for autoantibodies upregulated in pre-transplant sera of rejectors compared with sera of healthy controls calculated using SAM analysis. Table F, Fold change and q-value for non-HLA antibodies upregulated in post-transplant sera of AMR patients compared to non-AMR as calculated using SAM analysis. Table G, Fold change and q-value for non-HLA antibodies upregulated in post-transplant sera of AMR patients compared to non-AMR as calculated using SAM analysis (cohort divided into two groups).
(PDF)

## Abbreviations

AMR: antibody-mediated rejection
CAV: cardiac allograft vasculopathy
cPRA: calculated panel-reactive antibody
DSA: donor-specific antibody
dsDNA: double-stranded DNA
Hsp27: heat shock protein 27
Hsp60: heat shock protein 60
ISHLT: International Society for Heart and Lung Transplantation
LVEF: left ventricular ejection fraction
MFI: median fluorescence intensity
SAM: significance analysis of microarrays
SLE: systemic lupus erythematosus
ssDNA: single-stranded DNA
VAD: ventricular assist device
