# Supplementary material for: Generation of Antigen Microarrays to Screen for Autoantibodies in Heart Failure and Heart Transplantation
Source: PLoS One. 2016 Mar 11;11(3):e0151224. doi: 10.1371/journal.pone.0151224 (PMC4788148; doi:10.1371/journal.pone.0151224)
Supplement: S1 File — Table A, Antigen List for First Study (rejector vs. non-rejector). Table B, Antigen List for Second Study (AMR vs. non-AMR). Table C, Sequence of G-protein Coupled Receptor Peptides. Table D, Fold change and q-value for autoantibodies upregulated in pre-transplant sera from rejectors compared with non-rejectors. Table E, Fold change and q-value for autoantibodies upregulated in pre-transplant sera of rejectors compared with sera of healthy controls calculated using SAM analysis. Table F, Fold change and q-value for non-HLA antibodies upregulated in post-transplant sera of AMR patients compared to non-AMR as calculated using SAM analysis. Table G, Fold change and q-value for non-HLA antibodies upregulated in post-transplant sera of AMR patients compared to non-AMR as calculated using SAM analysis (cohort divided into two groups). (PDF) [file pone.0151224.s001.pdf]

Table A. Antigen List for First Study (rejector vs. non-rejector). This study included 58 antigens shown below.

**Antigen**	**Origin**	**Manufacturer**
Actin (rabbit muscle)	Rabbit Muscle	Molecular Probes
Actin A3653	Bovine Muscle	Sigma
Aggrecan, A1960	Bovine Articular Cartilage	Sigma
Aldolase, Type X	Rabbit Muscle	Sigma
α elastin	Human	Dr. Keeley (University of Toronto)
α-actinin	Chicken Gizzard	Sigma
α-ketoglutarate dehydrogenase	Porcine Heart	Sigma
Artery Cytoplasmic Lysate	Human Artery Tissue	ProSci
Artery Lysate	Human Artery Tissue	ProSci
Artery Membrane Lysate	Human Artery Tissue	ProSci
angiotensin 2 receptor (AT1R peptide)	Peptide	GenScript
β1 adrenergic receptor (B1AR) peptide	Peptide	GenScript
β2 adrenergic receptor (B2AR) peptide	Peptide	GenScript
β2 Glycoprotein I	Recombinant human, His-tagged	Diarect
Cardiac myosin	Porcine Heart	Sigma
Cardiolipin C0563	Bovine Heart, sodium salt	Sigma
Cardiolipin C1649	Bovine Heart, solution in ethanol	Sigma
Collagen IV C5533	Human Placenta	Sigma
Collagen V C3657 (Sigma Type IX)	Human Placenta	Sigma
Collagen IV C7521 (Sigma Type VI)	Human Placenta	Sigma
Collagen I C7774 (Sigma Type VIII)	Human Placenta	Sigma
Collagen III C4407 (Sigma Type X)	Human Placenta	Sigma
Copper Oxidized human LDL	Human Plasma	Academy Bio-Medical Company
Desmin	Recombinant Human	GenWay
dsDNA (genomic)	Salmon Testes, sodium salt	Sigma
EBNA-1	Recombinant	Biodesign
Enolase	Rabbit muscle	Sigma
Fibrinogen, type I	Human Plasma	Sigma
Fibrinogen, type I-S	Bovine Plasma	Sigma
Fibrinogen, type IV	Bovine Plasma	Sigma
GBM, dissociated	Recombinant human, His-tagged	Diarect

GBM, undissociated	Recombinant human, His-tagged	Diarect
GRP78 (HSPa5 or BiP)	Recombinant hamster	Enzo (Stressgen)
HBcAg	Recombinant	Sigma
Heart Cytoplasmic Lysate	Human Heart Tissue	ProSci
Heart Lysate	Human Heart Tissue	ProSci
Heart Membrane Lysate	Human Heart Tissue	ProSci
Heparin Sulfate	Bovine Kidney, sodium salt	Sigma
HSP 27	Recombinant Human	Enzo (Stressgen)
HSP 47	Recombinant Human	Enzo (Stressgen)
HSP 60	Recombinant Human	Enzo (Stressgen)
HSP 70	Recombinant Human	Enzo (Stressgen)
HSP 90	Native Human, from HeLa Cells	Enzo (Stressgen)
Human IgG	ChromPure human IgG, whole molecule	Jackson Immunoresearch
Insulin	Human	Sigma
Laminin	Engelbreth-Holm-Swarm murine sarcoma basement membrane	Sigma
Muscarinic M2 receptor M2R peptide	Peptide	GenScript
Myosin, calcium activated, M1636	Rabbit Muscle	Sigma
N2RA peptide	Peptide	Stanford
Proteoglycan	Bovine Nasal Septum	Sigma
Pyruvate dehydrogenase	Porcine Heart	Sigma
Ribo P (Ribosomal Phosphoprotein P0)	Recombinant human, His-tagged	Diarect
ssDNA	Calf Thymus	Sigma
Tropoelastin	Human	Dr. Keeley (University of Toronto)
Tropomyosin	Porcine Muscle	Sigma
Troponin I	Human Heart	Sigma
Troponin T	Human Heart	Sigma
Vimentin (Assay Designs)	Recombinant Human	Assay Designs

Table B. Antigen List for Second Study (AMR vs non-AMR). These studies were performed with a newer array with 64 antigens including more cardiac specific proteins.

**Antigen**	**Origin**	**Manufacturer**
α1A adrenergic receptor (A1AR) peptide	Peptide	GenScript
Actin (rabbit muscle)	Rabbit Muscle	Molecular Probes
Actin A3653	Bovine Muscle	Sigma
Aggrecan, A1960	Bovine Articular Cartilage	Sigma
Aldolase, Type X	Rabbit Muscle	Sigma
α elastin	Human	Dr. Keeley (University of Toronto)
α-actinin	Chicken Gizzard	Sigma
αβ-crystallin	Recombinant Human, His-tagged	Enzo (stressgen)
α-ketoglutarate dehydrogenase	Porcine Heart	Sigma
angiotensin 2 receptor (AT1R peptide)	Peptide	GenScript
β1 adrenergic receptor (B1AR) peptide	Peptide	GenScript
β2 adrenergic receptor (B2AR) peptide	Peptide	GenScript
β2 Glycoprotein I	Recombinant human, His-tagged	Diarect
Cardiac myosin	Porcine Heart	Sigma
Cardiolipin C0563	Bovine Heart, sodium salt	Sigma
Cardiolipin C1649	Bovine Heart, solution in ethanol	Sigma
Collagen IV C5533	Human Placenta	Sigma
Collagen V C3657 (Sigma Type IX)	Human Placenta	Sigma
Collagen IV C7521 (Sigma Type VI)	Human Placenta	Sigma
Collagen I C7774 (Sigma Type VIII)	Human Placenta	Sigma
Collagen III C4407 (Sigma Type X)	Human Placenta	Sigma
Copper Oxidized human LDL	Human Plasma	Academy Bio-Medical Company
Desmin	Recombinant Human	GenWay
dsDNA (genomic)	Salmon Testes, sodium salt	Sigma
EBNA-1	Recombinant	Biodesign
Endothelial cell cytoplasmic lysate	Human blood vessel: artery tissue	ProSci
Endothelial cell membrane lysate	Human blood vessel: artery tissue	ProSci
Endothelial cell lysate (total) with Triton X-100 lysis buffer	Human coronary artery endothelial cells	Rao Lab (University Health Network, Toronto)

Endothelial cell lysate (total) with SDS lysis buffer	Human coronary artery endothelial cells	Rao Lab (University Health Network, Toronto)
Enolase	Rabbit muscle	Sigma
Fibrinogen, type I	Human Plasma	Sigma
Fibrinogen, type I-S	Bovine Plasma	Sigma
Fibrinogen, type IV	Bovine Plasma	Sigma
GBM, dissociated	Recombinant human, His-tagged	Diarect
GBM, undissociated	Recombinant human, His-tagged	Diarect
GRP78 (HSPa5 or BiP)	Recombinant hamster	Enzo (Stressgen)
Heparin Sulfate	Bovine Kidney, sodium salt	Sigma
HSP 27	Recombinant Human	Enzo (Stressgen)
HSP 40	Recombinant Human	Enzo (Stressgen)
HSP 47	Recombinant Human	Enzo (Stressgen)
HSP 60	Recombinant Human	Enzo (Stressgen)
HSP 70	Recombinant Human	Enzo (Stressgen)
HSP 90	Native Human, from HeLa Cells	Enzo (Stressgen)
Human IgG	ChromPure human IgG, whole molecule	Jackson Immunoresearch
Human IgM	ChromPure human IgM, myeloma	Jackson Immunoresearch
ICAM-1 Protein	Recombinant Human	ProSci
Insulin	Human	Sigma
Laminin	Engelbreth-Holm-Swarm murine sarcoma basement membrane	Sigma
Muscarinic M2 receptor M2R peptide	Peptide	GenScript
Measles antigen	Edmonston Strain ATCC #VR-24	Meridian
Myosin Binding Protein C	Purified Peptide	Dr. Sadayappan (Loyola University)
Myosin, calcium activated, M1636	Rabbit Muscle	Sigma
N2RA peptide	Peptide	Stanford
Proteoglycan	Bovine Nasal Septum	Sigma
Pyruvate dehydrogenase	Porcine Heart	Sigma
Ribo P (Ribosomal Phosphoprotein P0)	Recombinant human, His-tagged	Diarect
ssDNA	Calf Thymus	Sigma
Tropoelastin	Human	Dr. Keeley (University of Toronto)
Tropomyosin	Porcine Muscle	Sigma
Troponin C	Recombinant Human	CalBioreagents
Troponin I	Human Heart	Sigma
Troponin T	Human Heart	Sigma
Vimentin (Assay Designs)	Recombinant Human	Assay Designs

Vimentin (Fitzgerald)	Recombinant Human	Fitzgerald

Table C. Sequence of G-protein Coupled Receptor Peptides. All of these peptides were synthesized against the second extracellular loops of the receptors. Peptides were dissolved in PBS to a final concentration of 0.2mg/ml and stored at -80°C until use.

**Receptor Antigen**	**Peptide Sequence**
Human Beta 1 Adrenergic Receptor	HWWRAESDEARRCYNDPKCCDFVTNR
Human Beta 2 Adrenergic Receptor	HWYRATHQEAINCYANETCCDFFTNQ
Human Muscarinic 2 Receptor	VRTVEDGECYIQFFSNAAVTFGTAI
Human Alpha 1A Adrenergic Receptor	GWRQPAPEDETICQINEEPGYVLFSAL
Human Angiotensin II Type 1 Receptor	IHRNVFFIENTNITVCAFHYESQNSTL

Table D. Fold change and q-value for autoantibodies upregulated in pre-transplant sera from rejectors compared with non-rejectors. Antigens with q-value <5% as calculated by SAM are considered significant.

**Antigens**	**Fold Change**	**q-value**
HSP 27	3.21	0
Cardiac Myosin	1.95	0
HSP 60	3.04	0
Ribosomal Phosphoprotein P0	2.47	0
ssDNA	2.36	0
Collagen V	2.23	0
Troponin I	3.03	0
Collagen I	2.86	0
dsDNA	1.57	4.37

Table E. Fold change and q-value for autoantibodies upregulated in pre-transplant sera of rejectors compared with sera of healthy controls calculated using SAM analysis. Antigens with q-value <5% are considered significant.

**Antigens**	**Fold Change**	**q-value**
Troponin I	3.38	0
Aldolase	5.78	0
HSP 60	4.37	0
Cardiac Myosin	2.14	0

Table F. Fold change and q-value for non-HLA antibodies upregulated in post-transplant sera of AMR patients compared to non-AMR as calculated using SAM analysis. Antigens with q-value <5% are considered significant.

**Antigens**	**Fold Change**	**q-value**
IgM IgG (Rheumatoid factor)	2.32	0
IgM Ribosomal Phosphoprotein P0	2.03	0
IgM Tropomyosin	2.73	0
IgM Oxidized human LDL	1.93	0
IgM ssDNA	2.41	0
IgM Endothelial Lysate Total	1.93	0
IgM dsDNA	2.19	0
IgG Cardiac Myosin	1.63	15.78

Table G. Fold change and q-value for non-HLA antibodies upregulated in post-transplant sera of AMR patients compared to non-AMR as calculated using SAM analysis (cohort divided into two groups).

Group 1

Antigens	Fold Change	q-value
IgM ssDNA	4.10	14.37
IgG GRP 78	3.52	14.37
IgM Endothelial Lysate Cytoplasmic	2.02	14.37
IgM Ribosomal Phosphoprotein P0	2.83	14.37
IgM Endothelial Lysate Total	2.72	14.37
IgM dsDNA	3.24	14.37
IgG IgM	1.96	14.37
IgM IgG (Rheumatoid factor)	2.42	16.76

Group 2

Antigens	Fold Change	q-value
IgM Tropomyosin	2.70	0
IgM IgG (Rheumatoid factor)	2.18	23.62
IgM Ribosomal Phosphoprotein P0	1.62	23.62
IgM Desmin	2.55	23.62
IgM Collagen, Type IV	1.49	23.62
IgM Alpha elastin	1.71	23.62
IgM Oxidized human LDL	1.76	23.62
IgM GRP 78	1.48	23.62
IgM Collagen, Type III	1.26	23.62
IgG Alpha-ketoglutarate dehydrogenase	1.40	23.62
IgM Laminin	1.29	23.62
IgM HSP 40	1.27	23.62
IgM Endothelial Lysate Total	1.43	23.62
